# 1,25-Dihydroxyvitamin D3 Inhibits the RANKL Pathway and Impacts on the Production of Pathway-Associated Cytokines in Early Rheumatoid Arthritis

**DOI:** 10.1155/2013/101805

**Published:** 2013-04-22

**Authors:** Jing Luo, Hongyan Wen, Hui Guo, Qi Cai, Shuangtian Li, Xiaofeng Li

**Affiliations:** ^1^Division of Rheumatology, Department of Medicine, The Second Hospital of Shanxi Medical University, Taiyuan, Shanxi 030001, China; ^2^Department of Medicine, The Second Hospital of Shanxi Medical University, Taiyuan, Shanxi 030001, China; ^3^Arizona Health Sciences Center, 1501 N. Campbell, Room 4104, P.O. Box 245051, Tucson, AZ 85724, USA; ^4^University of Washington, 1410 NE Campus Parkway, 459 Schmitz Hall, P.O. Box 355832, Seattle, WA 98195-5832, USA

## Abstract

*Objectives*. To study effects of 1,25-dihydroxyvitamin D3 (1,25(OH)_2_D_3_) on RANKL signaling pathway and pathway-associated cytokines in patients with rheumatoid arthritis (RA). *Methods*. Receptor activator of nuclear factor-kappa B ligand (RANKL), osteoprotegerin (OPG), IFN-**γ**, IL-6, TNF-**α**, IL-17, and IL-4 were examined in 54 patients with incipient RA using a cytometric bead array (CBA) or an enzyme-linked immunosorbent assay (ELISA). *Results*. After 72 hours of incubation of peripheral blood mononuclear cells (PBMCs) with 1,25(OH)_2_D_3_ in RA patients, the levels of RANKL, TNF-**α**, IL-17 and IL-6 significantly decreased compared to those of the control. 1,25(OH)_2_D_3_ had no significantly impact on the levels of OPG, RANKL/OPG, and IL-4. *Conclusions*. The present study demonstrated that 1,25(OH)_2_D_3_ reduced the production of RANKL and the secretion of TNF-**α**, IL-17, and IL-6 in PBMCs of RA patients, which indicated that 1,25(OH)_2_D_3_ might be able to decrease damage of cartilage and bone in RA patients by regulating the expression of RANKL signaling pathway and pathway-associated cytokines.

## 1. Introduction

Rheumatoid arthritis (RA) is a common chronic autoimmune disorder characterized by synovial inflammation. Bone loss in the inflamed joints [1, 2] occurs in the early stage of the disease, followed by the destruction of articular cartilage and bones. During the processing of the disease, the signaling pathway of receptor activator of nuclear factor kappa-B ligand (RANKL) and osteoprotegerin (OPG) is crucial in osteoclasts differentiation and activation [3].

An abnormal proliferation of T lymphocytes is a characteristic of RA. Previous data indicate that the accumulation and proliferation of T lymphocytes occurred prior to bone destruction [4, 5]. T lymphocytes play a role in differentiation and maturation of osteoclasts [6]. T lymphocytes secrete soluble cytokines such as RANKL, macrophage colony-stimulating factor (M-CSF), and tumor necrosis factor-*α* (TNF-*α*) and thereby directly induce the formation and differentiation of osteoclasts (direct effect) [7–9]. In addition, T lymphocytes produce interleukins such as IL-1, IL-6, and IL-17, which are absorption-promoting cytokines stimulating the expression of RANKL on the cell surface of mature osteoclasts, mesenchymal cells, or fibroblasts [10–12].

Subsequently, binding of RANKL to its specific receptor, receptor activator of nuclear factor kappa-B (RANK) on the surface of preosteoclasts, further increases differentiation, and maturation of osteoclasts [12]. Therefore, the published data suggest a close correlation between the RANKL pathway and joint deterioration in RA patients [13]. 

It is well known that 1,25-dihydroxyvitamin D3 (1,25(OH)_2_D_3_) plays an important role in the bone formation [14]. Currently, studies have suggested that 1,25(OH)_2_D_3_ is also an important immune modulator [15]. It has been demonstrated that 1,25(OH)_2_D_3_ directly inhibits T-cell proliferation and reduces its secretion of IL-2 and IFN-*γ* [16]. However, it is unclear whether 1,25(OH)_2_D_3_ is involved in the regulation of RANKL signaling pathway.

In the present study, using RA patients and healthy control peripheral blood mononuclear cells (PBMCs), we studied effects of 1,25(OH)_2_D_3_ on RANKL signaling pathway and associated cytokines. Methotrexate (MTX) was a common drug used in the treatment of RA because of its role of immune modulation [17]. Therefore, the effects of combination of 1,25(OH)_2_D_3_ and MTX on the RANKL signaling pathway as well as associated cytokines were also investigated. 

## 2. Materials and Methods

### 2.1. Subjects

54 incipient RA patients were recruited from the department of Rheumatology of the Second Hospital of Shanxi Medical University, including 18 males and 36 females with an age between 30 to 65 years old. They all fulfilled the American College of Rheumatology revised criteria for RA [18]. None of the patients had ever used vitamin D, glucocorticoids, immunosuppressants, or a tumor necrosis factor antagonist prior to the study. All patients had normal liver and kidney functions. 18 healthy volunteers were used as healthy control, and gender and age were completely matched to the RA patients. This study was approved by the Research Ethics Committee of the Second Hospital of Shanxi Medical University.

### 2.2. Sample Collection

18 mL peripheral venous blood was collected from fasting subjects in the early morning. 15 mL was placed in a tube with heparin sodium anticoagulant for extracting the peripheral blood mononuclear cells (PBMCs), and the remaining 3 mL for extracting serum was placed in a tube without any anticoagulant. The blood samples without anticoagulant were kept at room temperature for 30 minutes to allow coagulating followed by centrifuging for 15 min at 1,000 rpm. After centrifugation, the supernatants (serum) were removed and stored at −80°C for future experiments. 

### 2.3. In Vitro Stimulation and PBMCs Culture

Lymphocytes were isolated by density centrifugation from a 15 mL peripheral blood sample containing sodium heparin. Trypan blue staining was used to confirm that cell viability was >95%. The cells were suspended in phenol red-free Iscove's modified Dulbecco's medium (IMDM, Gibco, USA) supplemented with 10% charcoal-treated FCS, 100 units/mL penicillin, and 100 *μ*g/mL streptomycin, and the cell suspension was prepared at a density of 2 × 10^6^/mL. 

The healthy control and RA patients PBMCs were plated in a 96-well plate at 200 *μ*L/well and then treated with either vehicle (no stimulant) or the combination of anti-CD3 and anti-CD28 antibody plus 1,25(OH)_2_D_3_ at various concentrations (D1 = 0.1 nM; D2 = 1 nM; D3 = 100 nM), MTX at various concentrations (M1 = 0.05 ug/mL; M2 = 0.5 ug/mL; M3 = 1 ug/mL), or with the combination of 1,25(OH)_2_D_3_ and MTX (D2M2 group). 1,25(OH)_2_D_3_ and/or MTX treatment was performed only in anti-CD3 and anti-CD28 antibody treated cells. For the vehicle control, no stimulant was added to the wells, which meant that anti-CD3, anti-CD28, MTX, and 1,25(OH)_2_D_3_ cannot be added to PBMCs. The final concentration of anti-CD3 was 300 ng/mL and of anti-CD28 was 400 ng/mL. For cells treated with 1,25(OH)2D3 and/or MTX, the cells were treated with 1,25(OH)_2_D_3 _ and/or MTX plus anti-CD3 and anti-CD28 in a humidified, stable-temperature incubator at 37°C with 5% CO_2_ 72 hours after incubation, and the cultures were harvested by centrifuging at 2000 rpm for 8 minutes. The supernatants were collected and stored at −80°C for subsequent cytokines determination.

### 2.4. Measurement of RANKL, OPG, and Associated Cytokines in the Serum and Cell Culture Supernatant

The levels of RANKL and OPG were measured using ELISA (R&D Co, Ltd.). Analysis of IFN-*γ*, IL-4, IL-6, TNF-*α*, and IL-17 was conducted using a CBA human Th1/Th2/Th17 cytokine kit (BD Co, Ltd) and analyzed on a BDFACSCalibur flow cytometer. Quantity (pg/mL) of respective cytokine was calculated using CBA software.

## 3. Statistical Analyses

SPSS13.0 software was used for data analyses. All results were presented as mean ± standard deviation (M ± SD). All data met the conditions for a normal distribution and homogeneity of variance. To compare two groups of data, a completely randomized, independent, two-sample *t*-test was used; to compare multiple groups of data, a one-way ANOVA method of square-deviation was applied, and either the Student-Newman-Keuls (SNK) test or the rank sum test was used to compare data among the groups. *P*  value < 0.05 was considered to be significant.

## 4. Results

### 4.1. The Comparison of Serum Levels of RANKL, OPG and Associated Cytokines in RA Patients versus Healthy Control

We examined the expression of RANKL, OPG, and associated cytokines in the serum of RA patients and healthy control. Overall, there was a significant increase in RANKL, IL-17, IL-6, and TNF-*α* of RA patients when compared with those of healthy control (Table 1). Although OPG and RANKL/OPG showed a little increase in RA patients, no significant difference was observed. Further, the level of IL-4 was not significantly higher compared to that in healthy control. 

### 4.2. The Levels of Anti-CD3 Plus Anti-CD28 Induced RANKL, OPG, and Associated Cytokines in the Culture Supernatant of RA and Healthy Control PBMCs

 Anti-CD3/CD28 is the activator of T lymphocytes, and our data revealed that PBMCs of RA and healthy control cultured from freshly collected peripheral blood responded to the stimulation of anti-CD3 and anti-CD28 very well. The healthy control group and RA patients PBMCs were divided into vehicle control group and anti-CD3/CD28 group. In both RA and healthy control, after 72 hours stimulation, the levels of RANKL, TNF-*α*, IL-17, IL-6, and IL-4 in the anti-CD3/CD28 group significantly enhanced compared with the vehicle control group (*P* < 0.05; Table 2); although the level of OPG and RANKL/OPG in anti-CD3/CD28 group showed a little increase, the differences did not reach significance (*P* > 0.05, Table 2). 

### 4.3. The Levels of RANKL, OPG, and Associated Cytokines in the Culture Supernatant of RA Patients and Healthy Control' PBMCs Treated with MTX

**Figure 1:**
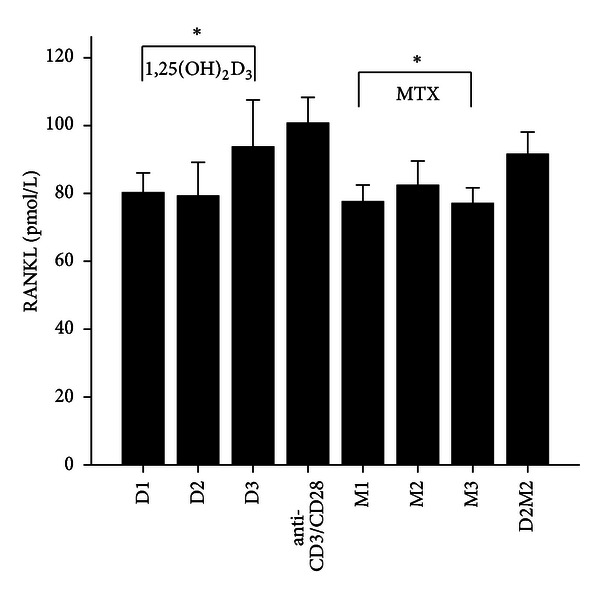
The levels of RANKL after treatment with 1,25(OH)_2_D_3_, MTX, and 1,25(OH)_2_D_3_ plus MTX in RA patients. The RA patients' PBMCs are treated with either anti-CD3/CD28, or 1,25(OH)_2_D_3_, MTX at various concentrations, or the combination of 1,25(OH)_2_D_3_ and MTX (D2M2 group). The levels of RANKL were detected and significantly decreased in the groups of 1,25(OH)_2_D_3_  and MTX compared to those of the group of anti-CD3/CD28 (*P* < 0.05). There was no difference in RANKL expression between the group of D2M2 and the group of Anti-CD3/CD28. *Mean *P* < 0.05.

**Figure 2:**
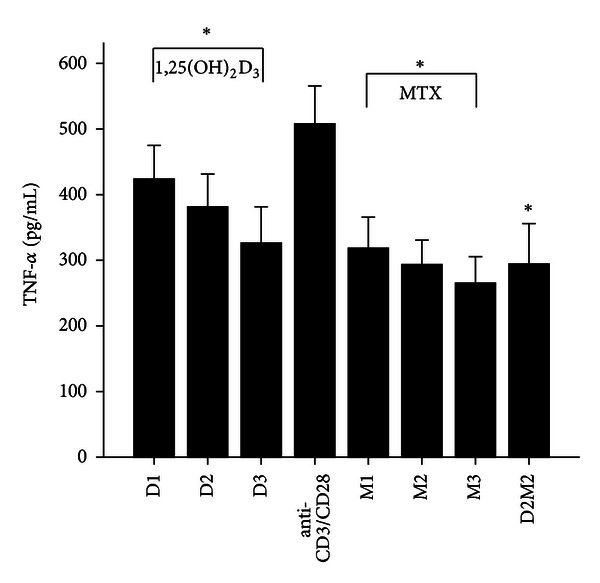
The levels of TNF-*α* after treatment with 1,25(OH)_2_D_3_, MTX, and 1,25(OH)_2_D_3_ plus MTX in RA patients. The RA patients' PBMCs treated with either anti-CD3/CD28, or 1,25(OH)_2_D_3_, MTX at various concentrations, or the combination of 1,25(OH)_2_D_3_ and MTX (D2M2 group). The level of TNF-*α* was detected and significantly decreased in the groups of 1,25(OH)_2_D_3_, MTX and D2M2 compared to the level in the group of anti-CD3/CD28 (*P* < 0.05). *Mean *P* < 0.05.

**Figure 3:**
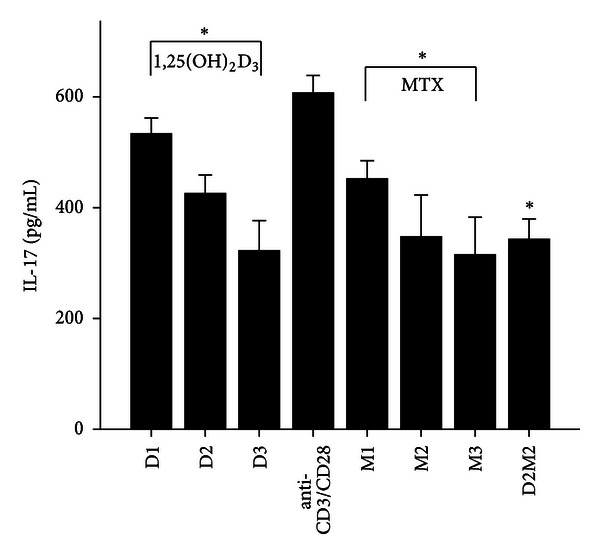
The levels of IL-17 after treatment with 1,25(OH)_2_D_3_, MTX, and 1,25(OH)_2_D_3_ plus MTX in RA patients. The RA patients' PBMCs are treated with either anti-CD3/CD28, 1,25(OH)_2_D_3_, MTX at various concentrations, or with the combination of 1,25(OH)_2_D_3_ and MTX (D2 M2 group). The RA patients' PBMCs treated with either Anti-CD3/CD28, or 1,25(OH)_2_D_3_ and MTX at various concentrations, or the combination of 1,25(OH)_2_D_3_ and MTX (D2M2 group). The levels of IL-17 were detected and significantly decreased in the groups of 1,25(OH)_2_D_3_, MTX and D2M2 compared to those of the group of anti-CD3/CD28 (*P* < 0.05). *Mean *P* < 0.05.

**Figure 4:**
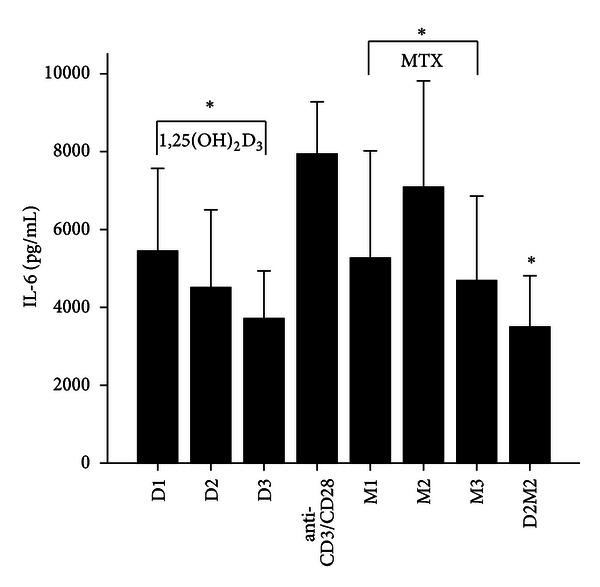
The levels of IL-6 after treatment with 1,25(OH)_2_D_3_, MTX, and 1,25(OH)_2_D_3_ plus MTX in RA patients. The RA patients' PBMCs are treated with either anti-CD3/CD28, or 1,25(OH)_2_D_3_, MTX at various concentrations, or the combination of 1,25(OH)_2_D_3_ and MTX (D2M2 group). The levels of IL-6 were detected and significantly decreased in the groups of 1,25(OH)_2_D_3_, MTX, and D2M2 compared to those of the group of anti-CD3/CD28 (*P* < 0.05). *Mean *P* < 0.05.

**Figure 5:**
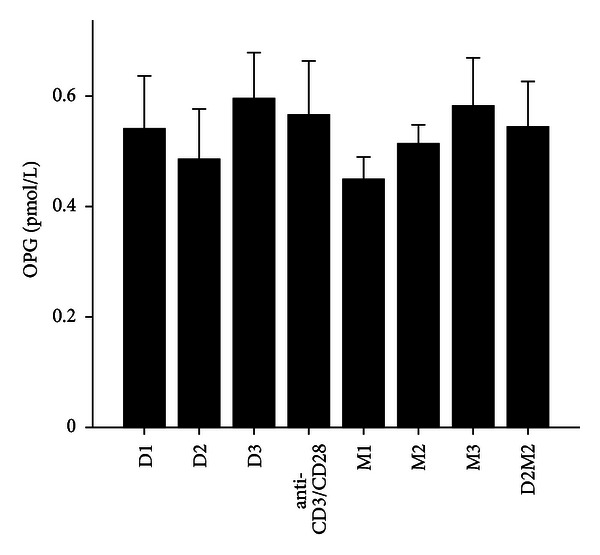
The levels of OPG after treatment with 1,25(OH)_2_D_3_, MTX, and 1,25(OH)_2_D_3_ plus MTX in RA patients. The RA patients' PBMCs are treated with either anti-CD3/CD28, or 1,25(OH)_2_D_3_ and MTX at various concentrations, or the combination of 1,25(OH)_2_D_3_ and MTX (D2M2 group). There was no difference in OPG expression between the groups of 1,25(OH)_2_D_3_, MTX, and D2M2 and the group of anti-CD3/CD28 (*P* > 0.05).

**Figure 6:**
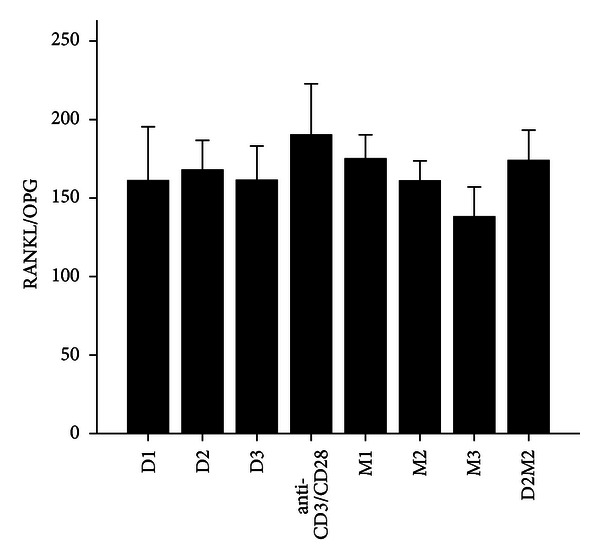
The levels of RANKL/OPG after treatment with 1,25(OH)_2_D_3_, MTX, and 1,25(OH)_2_D_3_ plus MTX in RA patients. The RA patients' PBMCs are treated with either anti-CD3/CD28, or 1,25(OH)_2_D_3_ and MTX at various concentrations, or the combination of 1,25(OH)_2_D_3_ and MTX (D2M2 group). There was no difference in RANKL/OPG expression between the groups of 1,25(OH)_2_D_3_, MTX, and D2M2 and the group of vehicle (*P* > 0.05).

**Figure 7:**
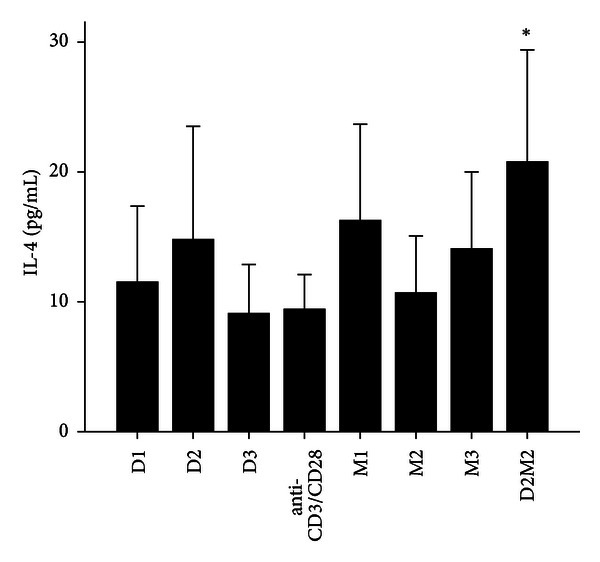
The levels of IL-4 after treatment with 1,25(OH)_2_D_3_, MTX, and 1,25(OH)_2_D_3_ plus MTX in RA patients. The RA patients' PBMCs are treated with either anti-CD3/CD28, 1,25(OH)_2_D_3_ and MTX at various concentrations, or the combination of 1,25(OH)_2_D_3_ and MTX (D2M2 group). 1,25(OH)_2_D_3_, MTX and 1,25(OH)_2_D_3_ plus MTX up-regulated the level of IL-4; however, there was no significant difference in IL-4 expression in the groups of 1,25(OH)_2_D_3_, MTX but there was significant difference in D2 M2 group (*P* < 0.05).

MTX had been demonstrated to be one of the most effective agents in current use for the treatment of patients with active RA [19]. Healthy volunteers and RA patients' PBMCs were divided into anti-CD3/CD28 group and three different MTX-dose-treated groups M1, M2, and M3. Our data revealed that 72 hours after incubation of PBMCs with MTX in RA patients, the levels of RANKL, TNF-*α*, IL-17 and IL-6 significantly decreased in MTX treated groups compared with Anti-CD3/CD28 group in RA patients (*P* < 0.05; Table 3; Figures 1, 2, 3, and 4). However, in three MTX treated groups, the inhibitions of pervious four cytokines were not in dose-dependent manner (*P* > 0.05; Table 3). The treatment of MTX had no significant effect on the levels of OPG, RANKL/OPG and IL-4 in MTX testing groups compared to those in anti-CD3/CD28 group in RA patients (*P* > 0.05; Table 3; Figures 5, 6, and 7). Further, in healthy control, there was no significant difference in all seven cytokines as mentioned above between the MTX-treated groups and anti-CD3/CD28 group (*P* > 0.05). 

### 4.4. The Levels of RANKL, OPG, and Associated Cytokines in the Culture Supernatant of RA Patients and Healthy Control' PBMCs Treated with 1,25(OH)_2_D_3_

To determine whether 1,25(OH)_2_D_3_ affected RANKL expression and associated cytokines, we tested three different doses of 1,25(OH)_2_D_3_ in anti-CD3/CD28-treated PBMCs of RA patients and healthy volunteers. 1,25(OH)_2_D_3_ treated groups were divided into D1, D2, and D3. Our data revealed that 72 hours after incubation of PBMCs with 1,25(OH)_2_D_3_ in RA patients, the levels of RANKL, TNF-*α*, IL-17 and IL-6 significantly decreased in 1,25(OH)_2_D_3 _ treated groups compared with anti-CD3/CD28 group (*P* < 0.05; Table 3; Figures 1, 2, 3, and 4). However there was no significant difference in previous mentioned four cytokines expression in three different dose groups and the inhibitions were not in dose-dependent manner (Table 4). The treatment of 1,25(OH)_2_D_3_ had no significant effect on the levels of OPG, RANKL/OPG and IL-4 compared to anti-CD3/CD28 group in RA patients (*P* > 0.05; Table 4; Figures 5, 6, and 7). Further, in healthy control, there was no significant difference in all seven cytokines as mentioned above between 1,25(OH)_2_D_3_-treated groups and anti-CD3/CD28 group (*P* > 0.05).

### 4.5. The Levels of RANKL, OPG, RANKL/OPG, TNF-*α*, IL-6 and IL-17 in the PBMCs Culture Supernatant of RA Patients and Healthy Control after Cotreatment with 1,25(OH)_2_D_3_ and MTX

To determine coeffect of 1,25(OH)_2_D_3_ and MTX, RA patients and healthy volunteers' PBMCs were divided into Anti-CD3/CD28 group and Anti-CD3/CD28+D2/M2 group. With the stimulation of anti-CD3/CD28, the cells were co-treated with MTX (M2) and 1,25(OH)_2_D_3_ (D2). Our data revealed that 72 hours after incubation of PBMCs with D2 M2 in RA, the levels of TNF-*α*, IL-17 and IL-6 significantly decreased compared with anti-CD3/CD28 group (*P* < 0.05; Table 5; Figures 2, 3, and 4) and the level of IL-4 in D2/M2 group significantly increased compared with Anti-CD3/CD28 group (*P* < 0.05; Table 5; Figure 7). Our data demonstrated that, there were no significant change in the levels of RANKL, OPG, RANKL/OPG in D2M2-treated group compared to those in the anti-CD3/CD28 group in RA (*P* > 0.05; Table 5, Figures 1, 5, and 6). Further, in healthy control, there was no significant difference in all seven cytokinesas mentioned above between the D2M2 treated group and Anti-CD3/CD28 group (*P* > 0.05).

## 5. Discussion

Rheumatoid arthritis (RA) is a common systemic autoimmune disease characterized by the destruction of articular cartilage and bone. Bone destruction was mediated by the multinucleated giant cells, osteoclasts. It has been shown that osteoclasts were responsible for deterioration of joint function in RA patients [20]. The augmentation of RANKL secretion is indispensable for osteoclast differentiation [3, 4]. The ligation of RANKL to its receptor, RANK, on the cytoplasm membrane of osteoclasts, causes bone resorption and destruction. In addition, RANKL also increases the survival of mature osteoclasts and enhances their function and consequently increases bone destruction [3, 21, 22]. In contrast, osteoprotegerin (OPG) is a soluble decoy receptor for RANKL by interfering with the RANKL/RANK binding, and it inhibits the maturation and activation of osteoclasts and their precursors [21, 22]. Therefore, it was very important to investigate RANKL expression and how to maintain the balance between RANKL and OPG in RA patients, which might provide an insight on the new treatment in reducing or preventing joint destruction in RA patients. Consistent with this idea, in the present study, we found that, in serum of RA patients, RANKL expression substantially increased compared to that in healthy control; however, OPG expression and OPG/RANKL ratio did not reduce significantly, which was not consistent with the other report by Kim et al. [23]. It was remaining controversial whether the serum levels of OPG and OPG/RANKL reflected what was happening in bone and joints in patients. Possible explanation was that OPG in the serum of such patients was bound to a plasma protein(s) and thus rendered inactive [13], and further studies will be required to determine the significance of these observations. We noted increased production of RANKL, TNF-*α*, IL-17, IL-6 and IL-4, following stimulation of the PBMCs with anti-CD3/anti-CD28, which suggested changes in the peripheral T-cell compartment. This finding indicated that the effect of anti-CD3/CD28 stimulation contributed to the increased cell activity in RA patients. 

There is an increasing appreciation that vitamin D exert broad regulatory effects on cells of the innate and adaptive immune system. These include reducing antigen presentation through reducing the activity of dendritic cells or promoting their tolerogenic phenotype, affecting the polarization of monocytoid cells, altering B cell function, decreasing chemokine gradients and reducing tissue-specific homing [24–27]. A significant literature in humans also indicates that vitamin D increases the activity of regulatory T cells to prevent the excessive activation of autoreactive T cells [28, 29].

Previous report identified that RANKL mRNA expression was inhibited by 1,25-dihydroxyvitamin [30]. Our study demonstrated that the effect of 1,25(OH)_2_D_3_ treatment on RANKL expression in the RA group reached significance, although there was no significant dose-dependent effect. In contrast, 1,25(OH)_2_D_3_ treatment did not have a significant effect on OPG levels or the RANKL/OPG ratio. Therefore, 1,25(OH)_2_D_3_ might either suppress the synthesis or decrease secretion of RANKL in PBMCs of RA patients. A previous study demonstrated that vitamin D might have clinical implications in the treatment of prostate cancer [31]. In addition, we tested the hypothesis that 1,25(OH)_2_D_3_ might show therapeutic effect in RA patients through the down regulation of RANKL expression, given that RANKL was also expressed in the synovial cells of RA patients, and the drugs might work differently in vivo versus in vitro. Therefore, in future studies, we will perform the effect of 1,25(OH)_2_D_3_ on RANKL expression in synovial cells and the extend the study.

Identically, methotrexate (MTX) is widely utilized for the treatment of patients with RA. MTX inhibits the expression of RANKL in RA patients in a dose-dependent manner, and also increases the secretion of OPG in RA supernatants [32]. In the present study, MTX treatment significantly decreased RANKL in the RA group. Although MTX decreased the expression of OPG and RANKL/OPG, this decline had no significant difference. Moreover, a higher MTX dose did not lead to a greater effect on the synthesis and secretion of RANKL, OPG or the RANKL/OPG ratio in patients' PBMCs. We postulated that MTX could effectively inhibit the synthesis and secretion of RANKL in RA patients' PBMCs. Cotreatment with 1,25(OH)_2_D_3_ + MTX reduced RANKL expression and the RANKL/OPG ratio; however, this reduction was not significant compared to MTX alone, indicating that further investigations were needed to determine the optimal dosage for both drugs. 

It is well known that the cytokine expression pattern is correlated closely between local and systemic inflammation as well as bone reabsorption and bone density loss. The authors [33, 34] find that IL-6, IL-17, and TNF-*α* intensify the inflammation response, worsen local joint synovial inflammation, and finally lead to the acceleration of joint cartilage destruction. Moreover, there is a synergistic effect of IL-17 and TNF-*α* [33], particulary, during the early phase of RA, the levels of these two cytokines are closely associated with joint deterioration. Therefore, these cytokines are involved in bone and cartilage damage in RA patients. The RANKL-RANK system, together with its endogenous inhibitor, OPG, perhaps represents the most important regulation in the interaction between bones and cytokines [21]. IL-17 has a strong catabolic effect by increasing osteoclast production directly as well as indirectly through an alteration in OPG/RANKL system from the osteoblasts [35]. The RANKL-mediated enhancement of calcification of smooth muscle cell in the coculture with bone-marrow-derived macrophage was dependent on TNF-*α* and IL-6 [36]. The evidence suggests that sIL-6R forms a complex with IL-6 that has been induced by TNF-*α* or IL-17, and that the resulting IL-6/sIL-6R complex induces RANKL expression [34]. The expression of RANKL is regulated by proinflammatory cytokines such as TNF-*α*, IL-6, and IL-17, which had demonstrated that the level of these cytokines is high in the serum and synovial fluid of RA patients [33, 34]. The literatures also suggest that these cytokines can induce RANKL expression, which breaks the balance between RANKL and OPG, increasing the differentiation of osteoclast progenitor cells into mature osteoclasts in mice model of collagen-induced arthritis [34–36]. Our present study revealed that 1,25(OH)_2_D_3_ had a significant impact on the expression levels of TNF-*α*, IL-17 and IL-6. These findings supported the idea that 1,25(OH)_2_D_3_ likely inhibited the expression of RANKL through reducing the synthesis and secretion of TNF-*α*, IL-17, and IL-6 in RA patients' PBMCs, which eventually decreased bone erosion. MTX and cotreatment with 1,25(OH)_2_D_3_ + MTX had the same significant impact on the expression of RANKL, TNF-*α*, IL-17, and IL-6. Our present study also suggested that 1,25(OH)_2_D_3_ or MTX treatment might affect the expression of RANKL or OPG through the inhibition of the aforementioned inflammation-associated cytokines and thus delay bone destruction. The further investigations will be needed to determine an optimal dose for each drug.

In summary, 1,25(OH)_2_D_3_ reduces whole-body bone loss and limits bone destruction in inflamed joints in RA patients. As an immunomodulatory drug, 1,25(OH)_2_D_3_ can be used in disease prevention without causing systemic immunosuppression. The present study demonstrates that 1,25(OH)_2_D_3_ reduces the production of RANKL and the secretion of TNF-*α*, IL-17, and IL-6 in PBMCs of RA patients, which indicates that 1,25(OH)_2_D_3_ might be able to decrease damage of cartilage and bone in RA patients by regulating the balance between proinflammatory and anti-inflammatory cytokines. Further studies are needed to be performed to test if 1,25(OH)_2_D_3_ directly affects the expression of RANKL and associated cytokines in cartilage or bone cells. 

## Figures and Tables

**Table 1:** The serum levels of RANKL, OPG, and associated cytokines in RA patients versus control group.

	RANKL (pmol/L)	OPG (pmol/L)	RANKL/OPG	TNF-*α* (pg/mL)	IL-17 (pg/mL)	IL-6 (pg/mL)	IL-4 (pg/mL)
RA group	100.17 ± 22.27	0.64 ± 0.17	169.57 ± 59.38	5.91 ± 2.53	42.56 ± 6.43	16.63 ± 12.00	2.72 ± 0.36
Healthy control group	75.82 ± 9.108	0.53 ± 0.16	149.00 ± 26.71	2.63 ± 0.27	21.10 ± 3.22	4.16 ± 2.27	2.72 ± 0.33

Values are expressed as mean ± standard deviation.

**Table 2:** The effect of Anti-CD3/CD28 induced the increases of inflammation-related cytokines in the PBMCs of RA and healthy control group.

	RANKL (pmol/L)	OPG (pmol/L)	RANKL/OPG	TNF-*α* (pg/mL)	IL-17 (pg/mL)	IL-6 (pg/mL)	IL-4 (pg/mL)
RA patient							
Vehicle control	85.39 ± 5.54	0.53 ± 0.13	171.10 ± 49.11	12.55 ± 5.32	46.23 ± 13.03	2884.35 ± 1389.03	4.53 ± 1.37
Anti-CD3/CD28	100.72 ± 11.98	0.57 ± 0.15	190.24 ± 51.25	508.52 ± 90.94	606.76 ± 49.79	7939.02 ± 2108.85	9.46 ± 4.15
Healthy control							
Vehicle control	67.22 ± 11.14	0.61 ± 0.18	127.18 ± 14.07	6.68 ± 0.55	25.48 ± 3.78	152.87 ± 304.38	5.02 ± 2.53
Anti-CD3/CD28	83.09 ± 12.17	0.61 ± 0.07	136.23 ± 13.42	195.95 ± 52.83	249.87 ± 17.63	2607.90 ± 232.98	9.77 ± 4.43

Values are expressed as mean ± standard deviation.

**Table 3:** The impact of MTX at various concentrations on inflammation-related cytokines in RA and healthy control group.

	RANKL (pmol/L)	OPG (pmol/L)	RANKL/OPG	TNF-*α* (pg/mL)	IL-17 (pg/mL)	IL-6 (pg/mL)	IL-4 (pg/mL)
RA patient							
Anti-CD3/CD28	100.72 ± 11.98	0.57 ± 0.15	190.24 ± 51.25	508.52 ± 90.94	606.76 ± 49.79	7939.02 ± 2108.85	9.46 ± 4.15
M1	77.60 ± 7.61	0.44 ± 0.05	175.62 ± 21.61	318.81 ± 74.45	451.50 ± 50.08	5255.36 ± 4309.03	16.30 ± 11.6
M2	82.57 ± 11.23	0.50 ± 0.06	164.74 ± 18.24	292.46 ± 58.67	372.13 ± 66.64	7251.50 ± 4455.93	10.73 ± 6.84
M3	77.12 ± 7.36	0.57 ± 0.13	139.14 ± 29.05	265.51 ± 64.08	315.10 ± 103.73	4706.41 ± 3391.34	14.13 ± 9.24
Healthy control							
Anti-CD3/CD28	83.09 ± 12.17	0.61 ± 0.07	136.23 ± 13.42	195.95 ± 52.83	249.87 ± 17.63	2607.90 ± 232.98	6.77 ± 4.43
M1	71.02 ± 16.39	0.45 ± 0.32	158.02 ± 38.89	161.43 ± 44.09	204.60 ± 21.31	1952.67 ± 355.35	15.57 ± 27.02
M2	71.41 ± 17.13	0.53 ± 0.06	134.89 ± 34.03	144.53 ± 24.13	188.03 ± 15.41	2177.13 ± 315.55	16.15 ± 11.55
M3	80.49 ± 24.10	0.57 ± 0.07	140.29 ± 42.54	128.42 ± 24.88	187.83 ± 41.34	3823.98 ± 2478.59	7.38 ± 2.82

Values are expressed as mean ± standard deviation.

**Table 4:** The impact of 1,25(OH)_2_D_3_ at various concentrations on inflammation-related cytokines in the RA and healthy control group.

	RANKL (pmol/L)	OPG (pmol/L)	RANKL/OPG	TNF-*α* (pg/mL)	IL-17 (pg/mL)	IL-6 (pg/mL)	IL-4 (pg/mL)
RA patient							
Anti-CD3/CD28	100.72 ± 11.98	0.57 ± 0.15	190.24 ± 51.25	508.52 ± 90.94	606.76 ± 49.79	7939.02 ± 2108.85	9.46 ± 4.15
D1	80.23 ± 9.37	0.53 ± 0.15	163.92 ± 56.07	424.08 ± 81.69	533.35 ± 47.47	5513.03 ± 3429.08	11.56 ± 9.14
D2	79.01 ± 15.41	0.48 ± 0.14	167.83 ± 29.43	381.56 ± 78.79	425.75 ± 55.33	4554.65 ± 3156.50	14.83 ± 13.65
D3	93.75 ± 21.88	0.58 ± 0.13	164.90 ± 35.68	326.18 ± 87.34	318.91 ± 85.91	3747.55 ± 1918.94	9.13 ± 5.88
Healthy control							
Anti-CD3/CD28	83.09 ± 12.17	0.61 ± 0.07	136.23 ± 13.42	195.95 ± 52.83	249.87 ± 17.63	2607.90 ± 232.98	6.77 ± 4.43
D1	82.43 ± 10.19	0.66 ± 0.12	129.67 ± 30.01	168.60 ± 50.01	219.48 ± 35.87	3229.37 ± 2029.54	7.27 ± 1.56
D2	15.46 ± 8.95	0.45 ± 0.11	175.07 ± 39.69	174.97 ± 26.36	211.48 ± 41.78	2601.70 ± 1032.23	9.47 ± 6.57
D3	74.67 ± 12.61	0.55 ± 0.09	139.44 ± 30.40	128.73 ± 19.29	206.53 ± 27.70	3236.45 ± 862.95	7.62 ± 3.31

Values are expressed as mean ± standard deviation.

**Table 5:** The impact of 1,25(OH)_2_D_3_ and MTX cotreatment on inflammation-related cytokines in the RA and healthy control group.

	RANKL (pmol/L)	OPG (pmol/L)	RANKL/OPG	TNF-*α* (pg/mL)	IL-17 (pg/mL)	IL-6 (pg/mL)	IL-4 (pg/mL)
RA patient							
Anti-CD3/CD28	100.72 ± 11.98	0.57 ± 0.15	190.24 ± 51.25	508.52 ± 90.94	606.76 ± 49.79	7939.02 ± 2108.85	9.46 ± 4.15
D2M2	91.60 ± 10.47	0.54 ± 0.13	174.64 ± 31.68	294.4 ± 97.24	341.53 ± 58.68	3464.63 ± 2061.39	20.82 ± 13.50
Healthy control							
Anti-CD3/CD28	83.09 ± 12.17	0.61 ± 0.07	136.23 ± 13.42	195.95 ± 52.83	249.87 ± 17.63	2607.90 ± 232.98	6.77 ± 4.43
D2M2	70.44 ± 13.01	0.52 ± 0.43	136.28 ± 27.89	151.48 ± 32.21	197.98 ± 43.97	2427.27 ± 238.13	10.73 ± 5.59

Values are expressed as mean ± standard deviation.
